# Application of Polymer Inclusion Membranes Doped with Alkylimidazole to Separation of Silver and Zinc Ions from Model Solutions and after Battery Leaching

**DOI:** 10.3390/ma13143103

**Published:** 2020-07-11

**Authors:** Elzbieta Radzyminska-Lenarcik, Malgorzata Ulewicz, Ilona Pyszka

**Affiliations:** 1 Faculty of Chemical Technology and Engineering, UTP University of Science and Technology, Seminaryjna 3 Street, PL 85-326 Bydgoszcz, Poland; ilona.pyszka@utp.edu.pl; 2 Faculty of Civil Engineering, Czestochowa University of Technology, Dabrowskiego 69 Street, PL 42-201 Czestochowa, Poland; malgorzata.ulewicz@pcz.pl

**Keywords:** polymer inclusion membrane, separation ions, silver, zinc, alkylimidazole

## Abstract

New materials, such as polymer inclusion membranes, can be used for water and wastewater treatment. In this paper, the selective transport of silver(I) and zinc(II) ions from nitrate solutions through the polymer inclusion membranes (PIMs), which consist of cellulose triacetate as a polymeric support, *o*-nitrophenyl pentyl ether as a plasticizer, and either 1-hexylimidazole (**1**) or 1-hexyl-2-methylimidazole (**2**) as an ion carrier, is studied. Both Zn(II) and Ag(I) model solutions (C_M_ = 0.001 M, pH = 6.5), as well as the solutions after the leaching of a spent battery with a silver–zinc cell (silver-oxide battery), are tested. The results show that Zn(II) ions are effectively transported through PIMs containing either carrier, whereas Ag(I) is more easily transported through PIMs doped with (**1**). In the case of the leaching solution after 24 h transport, the recovery coefficients of Ag(I) and Zn(II) for PIMs doped with (**1**) are 86% and 90%, respectively, and for PIMs doped with (**2**), 47% and 94%, respectively. The influence of basicity and structure of carrier molecules on transport kinetics is discussed as well. PIMs are characterized by using an atomic force microscopy (AFM) technique.

## 1. Introduction

The world’s silver reserves are estimated at approximately 540,000 Mg. Annually, approximately 22,000 Mg of this metal is extracted from ore. These data show that in approximately 25 years, silver reserves may be depleted. Therefore, the recovery of silver from various silver-containing waste materials, such as printed circuit boards, electronic devices, catalysts, and silver–zinc batteries, is necessary. Over the last 10 years, average annual silver recovery has constituted only 6600 Mg of silver, which is approximately 22% of the annual supply of this raw material [1]. This level varies greatly from country to country. For instance, in Sweden, Boliden produces approximately 150 Mg of silver from electronic waste (25% of its production) annually, whereas in the USA in 2015, only 21 Mg out of every 186 Mg of consumed silver was recycled, i.e., approximately 11%. The processing of waste materials is important as the largest producers obtain only 252 g of silver on average from 1 Mg of ore, and Boliden obtains 1 kg of silver on average from 1 Mg of waste mobile phones. Unfortunately, not all waste materials containing silver, including silver–zinc batteries, containing approximately 31% silver, are used efficiently. Annually, 1.5 billion silver–zinc batteries are produced, consuming almost 550 Mg of silver alone. Silver–zinc battery life does not exceed two years, so batteries become waste relatively quickly, and they need to be recycled for the recovery of silver. The level of recycling for this type of material is low. For instance, in Canada, only 2.2% of waste Zn–Ag batteries are recycled [2]. Therefore, effective waste management technologies are still being sought for this waste [3,4,5].

A number of reports on the recovery of silver from waste solutions (wastewater) containing silver can be found in the literature. Electrochemical methods [6], electro-flotation process [7], ion flotation [8,9], ion exchange [10], silica gel sorption on activated organosulfur compounds [11], chitosan-based hydrogel sorption [12], or adsorption [13,14] can be used to recover silver. Zinc can be also effectively recovered from waste materials by hydrometallurgical processes [15,16,17,18,19,20,21]. In hydrometallurgical processes, solid waste-containing metals, including silver and zinc, are comminuted and then leached [22,23]. For leaching silver, nitric acid is most commonly used [24]. Additionally, attempts were made to leach waste with thiourea and its derivatives [25]. The solutions obtained as a result of leaching are subjected to separation processes, which may include separation with liquid membranes [26,27,28,29,30,31,32,33,34]. Various carriers are used in membrane processes, e.g., thiourea derivatives [26,27], phosphoric acid derivatives [28,29], calixpyrroles [30,31], crown ethers [32,33,34,35,36] or calixarene [37]. Currently, polymer membranes are the most popular for membrane processes, which are increasingly used in the separation of various metal ions [38,39,40].

1-alkylimidazole (alkyl—from hexyl to decyl) were used for Cu separation from a Cu–Zn–Co–Ni mixture from nitrate [41,42] or chloride solutions [43], and for Zn separation from Zn–Co–Ni [44], Zn–Cd–Ni [45], and Zn–Mn mixtures [46]. 1-vinylimidazole was used to separate Cu(II) and Fe(III) ions during transport across polyvinyl acetate membranes [47]. 1-alkyl-2-methylimidazole (alkyl—from hexyl to octyl, and decyl) was used for Cu separation from a Cu–Zn–Co–Ni mixture [42,48,49,50], as well for Zn separation from a Zn–Cd–Ni mixture [45]. Separation of Zn from a Zn–Cd [51] or a Zn–Mn [45] mixture is also possible using alkyl imidazole derivatives.

The aim of this study was to examine the possibility of separation and recovery of silver(I) and zinc(II) from solutions after leaching of waste silver–zinc batteries in the process of transport through polymer inclusion membranes (PIMs). Alkyl imidazole derivatives of different structure, i.e., 1-hexylimidazole and 1-hexyl-2-methylimidazole, were used as ion carriers.

## 2. Materials and Methods

### 2.1. Reagents

**Figure 1:**
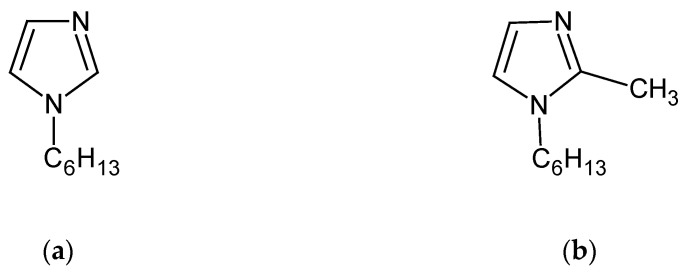
The chemical formula of 1-alkyl-imidazoles (carriers): (**a**) 1-hexylimidazole (**1**) b.p. 134–136 °C/12 mmHg; (**b**) 1-hexyl-2-methylimidazole (**2**) b.p. 130–131 °C/14 mmHg.

The inorganic chemicals, i.e., silver(I) and zinc(II) nitrates, 65% HNO_3_ solution, and tetramethylammonium hydroxide were of analytical grade and were purchased from POCh (Gliwice, Poland). The organic reagents, i.e., cellulose triacetate (CTA), *o*-nitrophenyl pentyl ether (*o*-NPPE), and dichloromethane were also of analytical grade, were purchased from Fluka (Busch, Switzerland), and were used without further purification. The 1-hexylimidazole (**1**) and 1-hexyl-2-methylimidazole (**2**) pictured in Figure 1 were synthesized according to the procedure described in [52].

The possibility of the separation of zinc and silver was tested from model solutions containing 0.001 mol/dm^3^ of the tested metal ions. Model solutions of each metal ion were prepared by dissolving appropriate amounts of nitrates in deionized water. All aqueous solutions were prepared using analytical reagent-grade chemicals and deionized water (conductivity = 0.10 μS/cm). Silver and zinc contents were determined using an atomic absorption spectroscopy (AAS) method (AAS Spectrometer, Solaar 939, Unicam, Geleen, Netherlands).

**Figure 2:**
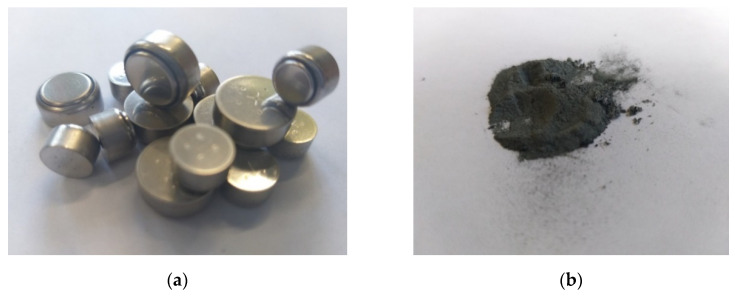
The button cell batteries (**a**) and used battery powder (**b**).

The spent silver-oxide (Zn–Ag_2_O) button cells (Figure 2) were obtained from a local market (watchmaking workshops). After mechanical comminution, the silver-oxide button cells (containing 10% zinc, 31% silver, 0.5% mercury, and other materials) were leached for 6 h with 2 mol/dm^3^ of HNO_3_ solution at a temperature of 70 °C. Under these conditions, 87.1% silver and 95.0% zinc were leached from the powder. The leached solution was then boiled with a small amount of activated carbon to remove any organic material. The obtained solution was filtered, and the levels of silver and zinc were determined using the AAS method. The Zn(II) and Ag(I) content was 9.5% and 27%, respectively. For transport tests, the solution was diluted.

### 2.2. Polymer Inclusion Membrane

The polymer inclusion membranes were obtained and prepared for metal ion transport according to the procedure described in previous studies [21,41,46,48,49,50,53]. The composition of the membranes and their thicknesses are given in Table 1.

### 2.3. Transport Studies

Transport experiments were carried out at the temperature of 20 ± 0.2 °C, according to the procedure described in previous papers [41,43,46,48,49,50,53,54]. The feed and receiving aqueous phases were an aqueous solution with pH = 6.5 (tetramethylammonium hydroxide) and 0.01 M HNO_3_, respectively. Changes in metal concentration in both phases were measured at appropriate time intervals.

## 3. Results

### 3.1. Characteristic of Membranes

**Figure 3:**
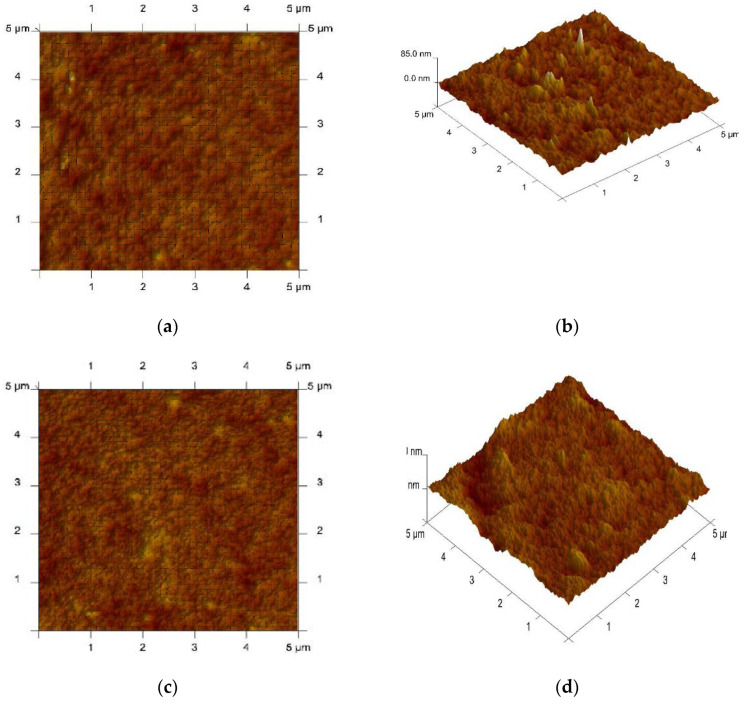
Atomic force microscopy (AFM) images (2D and 3D) of polymer inclusion membranes (PIMs) with alkylimidazol. (**a**,**b**) 1-hexylimidazole (**1**), (**c**,**d**) 1-hexyl-2-methylimidazole (**2**).

Surface PIM characterization was performed by atomic force microscopy (AFM) [21,41,43,46,53,54,55]. An AFM image of the PIMs doped with (**1**) or (**2**) in two- and three-dimensional forms is shown in Figure 3.

The darker areas in the AFM images (Figure 3) show elongated pores (cavity channels), which may indicate the crystallization of carrier molecules inside the CTA. The same effect was observed by Gherrou et al. [32].

Based on the membrane surface analysis (NanoScope v. 5.12 program, Park Systems Europe GmbH, Mannheim, Germany), the roughness (R_q_, nm) and the porosity (ε, [%]) were determined, which are shown in Table 2 with the tortuosity (τ), determined from the dependence (τ = 1 − ln ε), as developed by Wolf and Strieder [56].

The roughness, the effective size of pores, and the tortuosity have higher values for membranes containing (**2**) as the carrier. The roughness of the obtained membranes was lower than the roughness of the CTA membrane obtained by Tor et al., which was 14 nm [57]. The microstructure of the membrane (roughness [42,57,58], porosity [32,59], and tortuosity [56]) has an impact on the transport.

### 3.2. Transport of Zn(II) and Ag(I) Ions across PIMs from Zn–Ag Model Solution

Danesi [60] described the kinetics of transport via PIMs as a first-order process in relation to the metal ion concentration. Based on analytical data, the values of the permeability coefficient (P), initial flux (*J_o_*), selectivity coefficient (S), and recovery coefficient (RF) were calculated on the basis of relationships described in our earlier works [21,46]. All values given are mean values of three replicates with a standard deviation of 5%.

**Figure 4:**
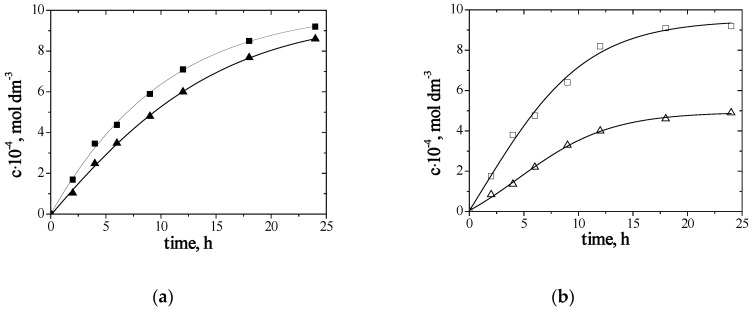
The changes of Zn(II) (■, □) and Ag(I) (▲, ∆) ions concentration over time during transport across PIMs with (**a**) (■, ▲) 1-hexylifiguremidazole (**1**) or (**b**) (□, ∆) 1-hexyl-2-methylimidazole (**2**).

The transport of Zn(II) and Ag(I) ions from equimolar nitrate solutions (pH = 6.5) through PIMs doped with (**1**) or (**2**) is discussed below. Two-component Zn–Ag model solutions containing metal ions at a concentration of 0.001 mol/dm^3^ were prepared for this study. The changes in Ag(I) and Zn(II) ions concentration during transport are presented in Figure 4.

**Figure 5:**
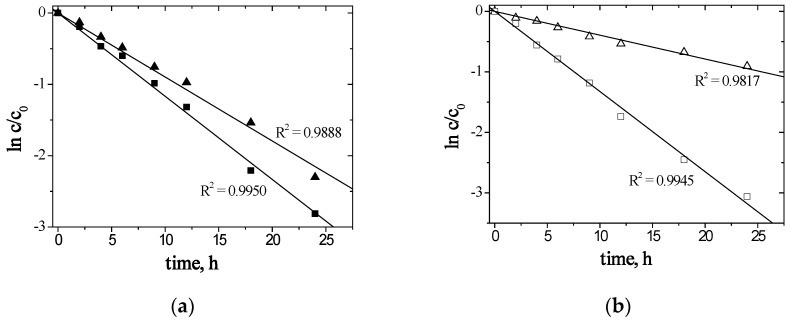
The relation of ln(*c/c_o_*) versus time for Zn(II) (■, □) and Ag(I) (▲, ∆) transport across PIMs with (**a**) (■, ▲) 1-hexylimidazole (**1**) or (**b**) (□, ∆) 1-hexyl-2-methylimidazole (**2**).

To calculate the value of the rate constant (*k*) for each membrane, relationships ln(*c*/*c_o_*) = f(t) were plotted (Figure 5).

In the case of the tested membranes, the functions ln(*c*/*c_o_*) = f(t) are rectilinear (the correlation coefficient (*R*^2^) ranging from 0.9817 to 0.9950), which confirms Danesi’s transport model [60]. The transport rate of metal ions decreased in the following order: Zn(II) > Ag(I), and though the transport rates of Zn(II) and Ag(I) ions through PIMs with (**1**) are similar, Ag(I) ions are much more slowly transported through PIMs doped with (**2**).

The values of parameters characterizing transport (initial flux values (*J*_0_) and selectivity coefficients S_Zn(II)/Ag(I)_) are shown in Table 3.

As indicated by the data shown in Table 3, for all investigated PIMs, the initial flux value for the transport of Zn(II) ions is higher than for Ag(I) ions and achieves the highest value for (**2**). In the case of a PIM doped with (**1**), both ions are transported and the Zn(II)/Ag(I) selectivity coefficient (S) has a lower value than (**2**), for which the coefficient is above four.

The literature [61,62,63,64] shows that the size and position of the alkyl group in alkylimidazole molecules affects both their acid-base and complex-forming properties. The stability constant values of Zn(II) and Ag(I) [62,63,64] complexes with the investigated carriers are summarized in Table 4.

Table 4 shows that the basicity of (**2**) is an order of magnitude higher than the basicity of 1-hexylimidazole (**1**). Complexes with Zn(II) ions have similar stability for (**1**) and (**2**). The stability of Ag(I) complexes is an order of magnitude higher for (**2**) than for (**1**). These highly complex-forming properties enable the use of (**1**) and (**2**) as effective carriers in PIM transport processes, and the difference in the stability of complexes forming in the membrane can be the basis for the separation of Ag(I) and Zn(II) ions.

The initial fluxes of Zn(II) ions are comparable in both carriers (Table 3), whereas the initial flux value for Ag(I) is much higher for (**1**) than for (**2**). The Zn/Ag selectivity coefficient (S) for (**2**) is much higher than for (**1**). The reason for this difference is the steric effect, resulting from the presence of a methyl substituent at position 2 in (**2**) (Figure 1). It hinders the formation of complexes with ions of many metals [46,49,53,54], and with Ag(I) ions in particular. The impact of the steric effect depends on the structure of the metal ion coordination sphere. In the case of zinc ions, tetrahedral complexes are formed next to octahedral complexes, facilitating the transport of Zn(II) ions through the membrane [46,49,53,54]. A similar phenomenon was observed in the case of Zn(II) separation from Zn–Cd–Co–Ni mixtures [65], Zn-Cd mixtures [51], Zn–Cd–Ni mixtures [45], and Zn–Mn mixtures [46], as well as zinc recovery from metallurgical waste [66] or galvanic wastewater [53].

### 3.3. Recovery of Metal

The recovery coefficients (*RF*) of Zn(II) and Ag(I) ions as a result of transport by PIMs doped with (**1**) or (**2**) from their equimolar nitrate solutions into HNO_3_ solutions are shown in Table 5.

In the case of (**1**), high and comparable recovery coefficients were obtained after 24 h transport, whereas in the case of (**2**), zinc recovery was very high (94%), but only 51% of the silver was recovered.

### 3.4. Transport of Zn(II) and Ag(I) Ions across PIMs from the Ag–Zn Battery Leaching Solution

**Figure 6:**
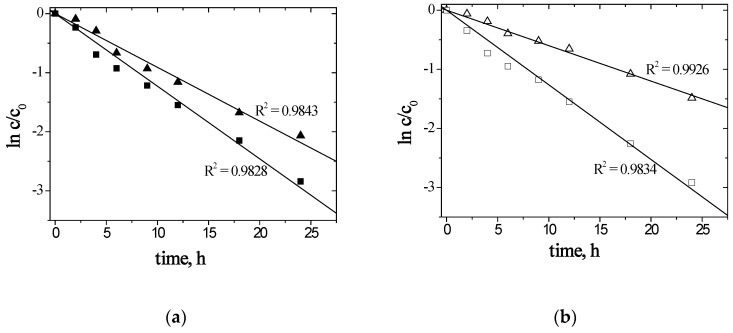
The relation of ln(*c/c_o_*) versus time from the leaching solution for Zn(II) (■, □) and Ag(I) (▲, ∆) transport across PIMs with (**a**) (■, ▲) 1-hexylimidazole (**1**) or (**b**) (□, ∆) 1-hexyl-2-methylimidazole (**2**).

The next step was the separation of Zn–Ag from the solution after leaching a battery with a silver–zinc cell. The concentration of silver(I) and zinc(II) ions in the solution was 0.003 mol/dm^3^ and 0.015 mol/dm^3^, respectively. In Figure 6, kinetic curves for transport of Ag(I) and Zn(II) ions from solutions after leaching the Zn–Ag_2_O battery are shown.

Figure 6 shows that ln(*c*/*c_o_*) dependencies as a function of time are linear. Hence, the transport of Ag(I) and Zn(II) ions through PIMs using (**1**) or (**2**), according to the model given by Danesi [60], can be described by the first-order kinetics in terms of the transported ions. The values of initial fluxes for the transport of metal ions from the solution after leaching waste batteries, a selectivity coefficient, and a degree of separation are shown in Table 6.

The initial flux, shown in Table 6, for Ag(I) ion transport through the polymer membranes containing (**1**) was higher than in the case of membranes containing (**2**). The initial flux for Zn(II) ion transport through the polymer membranes containing (**2**) was higher than in the case of membranes containing (**1**). The partition coefficients of Zn(II)/Ag(I) ions, using (**1**) or (**2**), were 1.13 and 3.46, respectively. The high recovery of zinc(II) (90%) and silver(I) (86%) was obtained as a result of the transport through membrane with (**1**). In the case of membrane with (**2**), the recovery of zinc(II) and silver(I) were 94% and 47%, respectively.

Comparing the data from Table 5 and Table 6, it can be concluded that the results obtained for model and true solutions are comparable.

## 4. Conclusions

The polymer inclusion membranes can be used due to their properties and long-term stability for water and wastewater treatment. The use of these polymer membranes containing 1-hexylimidazole (**1**) or 1-hexyl-2-methylimidazole (**2**) allows the separation of Ag(I) and Zn(II) ions in the transport process from both model and real solutions. In the case of PIMs with (**1**), both ions were transported and the Zn(II)/Ag(I) selectivity coefficient was not high. In the case of PIMs with (**2**), Zn(II) ions were transported better than Ag(I) ions, so the selectivity coefficient was above four. In the case of PIMs doped with (**1**), the recovery of both ions was high, both in the model solution and in the solution after leaching silver-oxide waste batteries. For PIMs doped with (**2**), the recovery of Zn(II) was almost double that of Ag(I), both in the model and real solutions. To separate Zn(II) and Ag(I) using a membrane technique, a two-stage process should be used: firstly, Zn(II) should be recovered using PIMs doped with (**2**); secondly, PIMs doped with (**1**) should be used to recover Ag(I).

## Figures and Tables

**Table 1:** The composition of the membrane used.

Support	Plasticizer	Carrier
cellulose triacetate (CTA)	*o*-nitrophenyl pentyl ether (*o*-NPPE)	1-hexylimidazole (**1**) or 1-hexyl-2-methylimidazole (**2**)
Quantitative composition: 2.6 cm^3^ *o*-NPPE/1 g CTA and 1.0 mol/dm^3^ carriers (calculated on plasticizer) Thickness: 28–31 μm (standard deviation below 1%)		

**Table 2:** AFM characterization parameters for PIM doped with 1-alkyl-imidazole.

Carrier in the CTA-*o*-NPPE Membrane	Effective Pore Size, µm	Tortuosity	Roughness (R_q_), nm
1-hexylimidazole (**1**)	0.050 ± 0.002	2.34	5.70 ± 0.05
1-hexyl-2-methylimidazole (**2**)	0.053 ± 0.002	2.37	6.20 ± 0.05

**Table 3:** The values of parameters characterizing Zn(II) and Ag(I) transport across PIM doped with alkylimidazole; membrane: pH of the feed phase was 6.5, receiving phase was 0.01 M HNO_3_.

Carrier	Metal Ions	Initial Flux *J*_0_, μmol/m^2^⋅s	Selectivity Coefficients S_Zn(II)/Ag(I)_
**1**	Zn(II)	2.02	Zn(II) > Ag(I)
	Ag(I)	1.46	1.38
**2**	Zn(II)	2.09	Zn(II) > Ag(I)
	Ag(I)	0.48	4.35

**Table 4:** The dissociation constants (pK_a_) of 1-hexylimidazole (**1**) and 1-hexyl-2-methylimidazole (**2**) and the stability constants (log *β*) their complexes with Zn(II) and Ag(I) ions.

Carrier	pK_a_ [56]	Metal Ions	log *β*
**1**	7.30	Zn(II)	5.87 [62]
		Ag(I)	6.33 [63]
**2**	8.32	Zn(II)	5.80 [64]
		Ag(I)	7.14 [63]

**Table 5:** The recovery coefficients (*RF*) of Zn(II) and Ag(I) ions from feed phase after 24 h transport across PIMs doped 1-hexylimidazole (**1**) or 1-hexyl-2-methylimidazole (**2**); conditions as in Table 2.

Carrier	Metal Ions	RF, %
**1**	Zn(II)	92
	Ag(I)	90
**2**	Zn(II)	94
	Ag(I)	51

**Table 6:** Initial fluxes (*J*_0_), selectivity coefficients (S), and recovery coefficients (*RF*) of Zn(II) and Ag(I) ions from feed phase after 24 h transport across PIMs doped with 1-hexylimidazole (**1**) or 1-hexyl-2-methylimidazole (**2**); feed phase: the leaching solution (pH = 6.5), receiving phase: 0.01 M HNO_3_.

Carrier	Metal Ions	*J*_0_, μmol/m^2^⋅s	S_Zn(II)/Ag(I)_	RF, %
**1**	Zn(II)	3.67	1.13	90
	Ag(I)	3.24		86
**2**	Zn(II)	3.98	3.46	94
	Ag(I)	1.15		47
